# Withering Minds: towards a unified embodied mind theory of personal identity for understanding dementia

**DOI:** 10.1136/medethics-2021-107381

**Published:** 2021-09-11

**Authors:** David M Lyreskog

**Affiliations:** 1 Department of Psychiatry, University of Oxford, Oxford, UK; 2 Wellcome Centre for Ethics and Humanities, Oxford, UK

**Keywords:** dementia, neuroethics, philosophical ethics, philosophy of medicine, applied and professional ethics

## Abstract

A prominent view on personal identity over time, Jeff McMahan’s ‘Embodied Mind Account’ (2002) holds that we cease to exist only once our brains can no longer sustain the basic capacity to uphold consciousness. One of the many implications of this view on identity persistence is that we continue to exist throughout even the most severe cases of dementia, until our consciousness irreversibly shuts down. In this paper, I argue that, while the most convincing of prominent accounts of personal identity over time, McMahan’s account faces serious challenges in explanatory power of dementias and related neurodegenerative conditions. Particularly, this becomes visible in the face of emerging methods for neural tissue regeneration, and the possibility of ‘re-emerging patients’. I argue that medical professionals’ neglecting qualitative aspects of identity risks resulting in grave misunderstandings in decision-making processes, and ethically objectionable outcomes in future practices. Finally, I propose revisions which could potentially salvage the great benefits that Embodied Mind Theory still can bring to the field of dementia care in terms of understanding life, death, and identity across the lifespan.

## Introduction

As we learn more about the nature of dementias and what we might do to prevent and treat them, the moral value of personal identity—of persisting through time as unique individuals—remains an enigma. There is no lack of literature either on personal identity and neurodegenerative disease,^1–8^ or on neurotechnological interventions and personal identity.^9–16^ Indeed, the debates in both of the above-mentioned fields are quite alive and well. Now, many times applied ethics literature works with a bottom-up approach, where observations and anticipations of real-life issues relating to identity are highlighted and/or analysed. Although there is nothing wrong with this approach per se—and indeed, great work has been done in this area—it oftentimes leads to a surprisingly low level of attention to robust theoretical work on what may be referred to as ‘traditional’ theories of personal identity. In this class of theories, one may count Brain Identity (BI) Theory^17 18^ and Psychological Continuity Theory.^19^ In these works, the focus of inquiry is the persistence of numerical identity over time. In recent years, another view on personal identity, often referred to as ‘the Narrative view’ has also gained ground, focusing more on ‘the continuation of an inner story’,^20^ rather than numerical identity of persons.

On the other hand, there is also a concern that applying the same frameworks to practice may lead to misunderstandings and ethically objectionable outcomes, as they do not accurately describe how dementia, care and emerging treatments for dementia may affect identity. The aim of the paper is to bridge the gap between prominent theoretical account on personal identity over time, and the philosophical and ethical realities of dementia care and treatment. In what follows, we will first briefly visit the qualitative/numerical identity distinction. Here, the idea that narrative identity relations are what matters will also be reviewed. It will be argued that narrativists make a good point in criticising the tunnel vision focus on numerical identity, but that their solution (a refocus towards narrative stories) is unsatisfying with regard to certain aspects important to our enquiry. Second, we will visit some of the more prominent schools of thought with regard to personal identity, including Brain Identity (BI) Theory, Psychological Continuity (PC) Theory and Embodied Mind (EM) Theory. Third, a modified EM account of personal identity will be argued for, which allows for a more intuitive understanding of what is going on in cases of neural decline and dementias, and in the treatments of the same.

## Qualitative and numerical identity, and the narrative view

One of the fundamental distinctions in theory of personal identity is that of qualitative identity and numerical identity.^1^ Parfit^19^ used the metaphor of billiard balls to distinguish between the two types of identity in a colourful way:

[T]wo white billiard balls are not numerically but may be qualitatively identical. If I paint one of these balls red, it will cease to be qualitatively identical with itself as it was. But the red ball that I later see and the white ball that I painted red are numerically identical. They are one and the same ball. We might say, of someone, ‘ After his accident, he is no longer the same person’ . This is a claim about both kinds of identity. We claim that *he*, the same person, is *not* now the same person. This is not a contradiction. We merely mean that this person’s character has changed. This numerically identical person is now qualitatively different. [Parfit, pp. 201–202]^19^

Simplistically put, one may say that numerical identity is the identity of *who* we are (as opposed to another individual), and qualitative identity is that of *how* we are. Now, while empirical work tends to focus on qualitative and narrative aspects of identity, theoretical philosophical work tends to focus on numerical identity: the persistence of a person as a specific individual. Arguably, we accept that we change (qualitatively) over time, as we live our lives, while to ‘change’ numerically is one of our greatest horrors—as it seems to mean that we cease to exist altogether. Therefore,concerns about personal identity as a moral value at stake tend to be about numerical identity.

In recent years, however, voices have been raised against this focus on numerical identity. Jecker and Ko,^20^ conceding that numerical identity does hold some value for certain things in life, argue:

We regard ourselves not merely as individuals persisting over time, but also as persons leading lives. Leading a life requires unifying events in our lives by selecting, interpreting, and weaving them into an intelligible plot, with beginning, middle, and closing chapters. We impart meaning, for both ourselves and those around us, by situating what we do and want within the context of a meaningful self-told story. A person’s narrative shows who that person is, and how that person understands his or her central desires, goals, and personality.[Jecker and Ko, p. 161]^20^

This critique from Jecker and Ko echoes the main standpoint of narrativist theory of personal identity: we should understand our identities as coherent, self-told stories.^21–23^ There is an idea behind the recent support of narrative identity theory that this is the kind of identity—let’s refer to it as ‘narrative identity’—we actually care about, and that it is also more practical: if a person feels that she can tell a coherent story about her life with which she identifies, identity is undisrupted.^24^

There are at least three reasons why this approach appears incorrect and/or insufficient for our purposes. First, and fundamentally, for there to be a coherent self-told story to be told, there must be a numerically identical person to tell it. If there is no person to tell the story, it can by definition not be self-told—because there is no self present. In the same fashion, if there is another person (numerically) telling the story, it cannot be self-told—because the story would not be about the self.^2^ In this way, it appears that if narrative identity matters, numerical identity should matter at least as much, as it is a necessary condition for there to be a narrative identity at all.^3^ In this indirect way, we should care about numerical identity if we care about narrative identity.

Second, numerical identity appears to matter also in a more direct manner. Consider a hypothetical case where our coherent story would be told by another person than ourselves. Just because someone thinks that they are us, and believe they are talking about their own life when telling our story, it doesn’t mean that they *are* us.^4^ It is also conceivable that we are unable to form a coherent story about our own lives: perhaps memories conflict, or have been blurred over the years, to a point where there is no ‘intelligible plot, with beginning, middle, and closing chapters’.^20^ Does this mean that we have ceased to exist? On the contrary, it seems—at least prima facie—that we can retain our identity even if our memories, dispositions and attitudes over time have become somewhat scrambled and distorted.

Third, although narrative identity accounts allow us to inquire with patients directly in matters regarding their identity, it is not clear that the approach is practical in the context of age-related neural decline and disease. We are often faced with cases where the patient is completely incapable of telling a true and coherent story about her life, or is only able to recount a very select set of sections from it—not seldomly scrambled and distorted—only to later on, often shortly before dying, display clarity and improved ability to access her past. These phenomena are highly problematic on most accounts, but perhaps even more so for narrative identity theory: is the identity relation broken, solely because the person cannot accurately tell a coherent story about herself at all times? Or can it be ‘on a break’ as it were?^25^ The simple (or, perhaps, simplistic) solution that would make the narrative approach practical—to use the response of the patient as the main evidence of identity—backfires immensely in cases like this, and more generally in dementia. For these reasons, a narrative approach is not sufficient to account for personal identity in the context of age-related neural decline and disease.

That being said, there appears to be something to the original narrativist critique, namely the traditional accounts’ focus on numerical identity alone, and dismissal of qualitative identity as a reliable indicator of identity in persons. Or, rather, the tendency to dichotomise the distinction between qualitative and numerical identity.^26^ Recalling the quote above from Parfit, [pp. 201–202]^19^ to say: ‘after his accident, he was no longer the same person’, is not a contradiction. It is not clear why there is a tendency in literature to claim that one kind of identity (usually numerical) is more important than the other. Furthermore, it is not clear that it makes much sense to separate the numerical identity (that someone is (not) numerically the same person) and qualitative identity (how someone is as a person) so clearly in cases of dementia. Rather, I will argue, it is vital for our understanding of identity in dementia that we seek to understand the relationship between numerical and qualitative identity.

Consider the following two sentences:

1. Lately, my father is no longer the same person.
2. That is no longer my father.

These are two sentences which could very well be taken from conversations with, say, the children of parents with progressive dementia. On the surface, the two sentences mean roughly the same thing. But if we truly take the phrasing seriously, there is an existential and ethically charged difference between them. One (1) appears to be about a numerically identical person whose qualitative identity has been severely altered. The other (2) seems to be about a person who is no longer numerically identical to a person who used to inhabit the same body. What is the most interesting for us here is not the phrasing itself—it is not a play of words. What is crucial here, is that the sentences are expressions of how the same process (progressive dementia) can result in distinct phenomena. Sometimes at different points in time, sometimes simultaneously but from different perspectives. But it is the same forms of decline, the same diseases, that eat away at both numerical and qualitative aspects of the identity of a person—at least on many accounts of personal identity. As McMillan puts it: ‘Given that dementia has a profound effect on an individual’s mental life, it is entirely possible they may change so much that it raises serious doubts about whether there exists the degree of psychological continuity and connectedness necessary for numerical personal identity.’^2^ We shall soon have a closer look at what this might mean for the purpose of defining personal identity in this context. For now, let us settle with the notion that, although narrativist accounts of personal identity highlight the problematic distinction between numerical and qualitative identity, they are not sufficiently helpful in addressing ethical issues related to personal identity in dementia care and treatment. Typically leaving the numerical aspects of identity downplayed or ignored, they risk to be misleading in decision-making processes and caregiver support. Let us look have at some other options.

## The strengths of Embodied Mind Theory

There are many accounts in the literature of personal identity over time, all with various advantages and disadvantages. Let us first distinguish between two of the main streams in contemporary philosophy of personal identity.

First, we have Psychological Continuity Theory.^5^ Having been around since at least the days of Locke^27^ in the form in which we know it today, PC Theory gained ground over the last few decades through the works of philosophers such as Shoemaker and Lewis,^28 29^ and was perhaps most famously championed by Parfit.^19^ It has grown to become one of the most widely supported approaches to understanding and theorising personal identity over time,^30^ and typically stresses the continuity of a specific psychology as the main factor in identity retention over time. We can formulate a generic account of personal identity over time according to psychological continuity theory like so: the persistence of a person over time requires that certain psychological relations hold for a specific person across a given amount of time. That is, person X at t1 is identical to person Y at t2 if and only if a certain relation P holds between X at t1 and Y at t2. Exactly what that psychological relation P must contain is a matter of dispute among proponents, but it commonly includes memories, attitudes and/or values.

Second, we have a family of theories which one may refer to as Brain Identity Theory.^6^ In this school of thought, what marks the continued identity of a person is the persistence of the ‘container’ of psychological content. The most prominent proponent of this view, on a theoretical level, is Nagel, but the view is arguably fairly prevalent in the neurosciences in general.^17 18 31^ We can formulate a generic account of personal identity over time according to BI Theory as saying: we are our brains, or certain parts of our brains, and our identity relies on the persistence of those (parts of our) brains over time.

Both BI Theory and PC Theory provide us with intuitively appealing expositions of how we might best understand persistence of identity over time. Starting with PC Theory, Parfit’s account is today arguably the most well known and frequently referred to. His criterion for personal identity over time is formulated as follows:

The Psychological Criterion: (1) There is *psychological continuity* if and only if there are overlapping chains of strong connectedness. X today is one and the same person as Y at some past time if and only if (2) X is psychologically continuous with Y, (3) this continuity has the right kind of cause, and (4) it has not taken a ‘ branching’ form. (5) Personal identity over time just consists in the holding of facts like (2) to (4). [Parfit, p. 207]^19^

There are several implications of this account, in the context of dementia, that are worth noting. First, persons typically fade away gradually and slowly, rather than suddenly and rapidly. In the case of Alzheimer’s disease, for instance, there seems to be no clear line where identity is lost or transformed into a new identity. At first glance, this could be problematic: one may think that either identity holds, or it does not (X=Y or X≠Y). But for an account subscribing to the psychological criterion, this is not necessarily a problem per se: we can explain the gradual process of diminishing or shifting identity by looking at the level of connectedness held between a person at different times. If we look at a large timespan, say, t1–t10, it may be that there is strong connectedness between the person at every point in time (A at t1=B at t2, B at t2=C at t6, etc), and yet it may seem that the person at t10 is vastly different—not to say another—than at t1 between A at t1 and J at t10. So, looking solely at the individual qualities over time, the identity of person A may appear to have been altered, broken, or lost, and yet there was no clear point in time when it happened. But this causes a series of conundrums. Most notably, dementias are typically not linear in their progression. It is not unusual for patients to be more connected to their previous selves on some days, and less so on others. What does this mean for their continued persistence? For Parfit, and for many other advocates of PC Theory, the fluctuation of identity is not a big problem. Parfit famously argued that personal identity is not what matters, so we should not care too much about it. What matters, according to Parfit, is the continuum of connected psychology (i.e., (1) and (2) of the psychological criterion).^19^

Although this is a neat way of avoiding theoretical problems related to PC Theory, it is not a very satisfactory one in practice. The idea that we should in principle not care whether we as individuals cease to exist and are gradually replaced by a person who will inhabit our organism thinking they are us, is arguably difficult to apply in practice. Imagine a dialogue between a caregiver/child (C) and a doctor (D) of a patient (P) suffering from late-stage AD dementia:

C: It is so strange… I can see that P is right there in front of me. And some days I can have a normal conversation with her. But other days, somehow, it does not feel as if she is the same person I used to know. My loving mother, growing up…

D: Oh, she is definitely not. This woman has not been your childhood mother—that person is long gone! Several times over, probably. But don’t worry—that doesn’t matter!

It would just be not only insensitive of a doctor to say such a thing, but also, it appears, incorrect. The swaying of identity in persons living with dementia is something different than the psychological changes we all go through over the course of a life, in that it appears to eat away at a person’s identity. And during this process of withering away, the maintenance and retention of that identity is at the core of our concerns. In this way, it seems that personal identity does matter. Furthermore, these examples suggest that there are certain aspects of identity, and identity change, which are more problematic than others.

BI Theory offers a different set of pros and cons. For Nagel, the most prominent defender of this strand of personal identity theory, there are no indeterminate states of identity—identity always either holds, or it does not, and our brain is solely the determinator of that relation. Or, more precisely, Nagel argues that identity is determined by a certain brain in a certain state:

If […] my mental life depends entirely on certain states and activities of my brain, […] then that brain in those states […] is what I am.[Nagel, p. 41]^31^

Contrary to Parfit, Nagel argues that personal identity matters quite a bit.^7^ Life, he argues, can be a wonderful thing—and even when it isn’t, death is worse, as it deprives the victim of any future goods. On Nagel’s account, then, the end of one’s personal identity means death. And if personal identity is determined by our brains, then our death occurs when our brain dies. In this straight-forward way, BI Theory appears fairly intuitive. Changes over time in psychology do not destabilise or erase our identity, so the theory avoids most arguments against Psychological Continuity Theory of personal identity over time.

However, it is not without gaps, particularly in the context of neurodegenerative disease and dementia. One of the main criticisms of Nagel’s BI account is that it does not specify what exactly it takes for me to survive: is it my whole brain?; My brain as it is at a given point in time?; Can another vessel do the job? In the appendix to Reasons and Persons, Parfit highlights this weakness of Nagel’s account: in a case where a person’s brain structure is ‘tampered with’ so that psychological connectedness is lost, it is not clear if she will survive or not, depending on how we interpret Nagel’s brain criteria. If, on the one hand, she is her brain as a whole, and if the brain survived the tampering she would still be alive. If, on the other hand, she is her brain in a certain state, she would plausibly not survive [Parfit, pp. 468–477].^19^ The key problem is that there is no requirement on a BI account for psychological content, mental states, or the presence a mind for us to continue to exist.

Without digging too deep into the debate between Parfit and Nagel, we can at this point see a clear issue for BI Theory in the context of dementia. First, it appears trivially true that we—as persons persisting through time—survive plenty of changes in our brain. Through plasticity, our brains grow and re-shape throughout life—not only in childhood, or as results of trauma. So, a BI theory should at the very least be able to accommodate for such mundane changes of brain structure. On the other hand, if the continued existence of our brain as a whole is what matters, BI Theory runs the risk of being too permissive: it would imply that a person is alive as long as the brain performs basic motor and life-support tasks, while (perhaps due to traumatic injury, or due to neurodegenerative disease) the brain has lost its capacity for consciousness. Consider yet again the following two phrases.

1. ‘Lately, my father is no longer the same person’.
2. ‘That is no longer my father’.

On an account like the latter, statement ‘2’ is nonsensical, unless the father’s brain has been destroyed or faced whole brain death. This could be a correct assessment, and one can certainly argue for such a position. A major drawback of such a move, however, is that we are forced to accept the ‘survival’ of persons who have irreversibly lost all capacity for an inner life with sensations, thoughts, or experience of actually being alive. While this may be a theoretically coherent position, such a theory seems to be of little practical use in the context of medical decision making. When it comes to the persistence of persons—as opposed to that of inanimate objects—we value the capacity for consciousness. This brings us to a third option. Carrying in mind the benefits and issues associated with both PC Theory and BI Theory, let us consider a third class of identity theory: Embodied Mind Theory. In particular, we shall have a look at the Embodied Mind Account (EMA), as presented by Jeff McMahan:

[ We ] are essentially embodied *minds*. […] According to the Embodied Mind Account, the criterion of personal identity is physical and functional continuity of the brain. [T]here need only be enough physical and functional continuity to preserve certain basic psychological capacities, particularly the capacity for consciousness. [McMahan, pp. 68–69]^7^

The EMA has gained a somewhat odd position within the literature on personal identity, in that it appears to not clearly align with any PC Theory or BI Theory, while it at the same time seems to be intimately related to both. On the one hand, what matters for personal identity is the minimal physical and functional continuity of those parts of the brain—whichever they may be—that are necessary and sufficient for maintaining consciousness.^8^ In this way, McMahan’s account is largely understood as related to Brain Identity accounts.^7 21 32 33^ ^9^ On the other hand, McMahan argues, while personal identity matters to some extent, what matters most is some kind of psychological unity in life, which generates a cause for ‘egoistic concern’: that is, to care about one’s future self as one cares about oneself, rather than as for another. Furthermore, McMahan makes the case that our personal identity and basis for egoistic concern largely coincide, as personal identity itself usually constitutes a sufficient cause for egoistic concern [McMahan, pp. 69–82].^7^ Also, it is worth recalling, we are after all embodied *minds*, where the ‘mind’-part arguably is constituted by psychological connectedness and continuity.

This layout gives EM Theory a powerful structure. Unlike many BI accounts, it narrows down which parts of the brain are necessary for the criteria to be satisfied (although vaguely). Additionally, the emphasis on our ‘selves’ as minds, in combination with the inclusion of psychological connectedness as what matters, does at least to some degree correspond to an intuition that our mental lives matter in identity. It also, unlike much of PC Theory, ties our minds and psychological continuity to a specific vessel (the consciousness-regulating parts of our brains), which avoids the improbable conclusion that identity potentially alters several times throughout a normal life. As such, the EMA, as presented by McMahan,^7^ serves as a strong candidate for a theoretical basis when analysing ethical issues related to dementia and identity. That being said, there are a few implications of the McMahan’s EMA that appear counterintuitive at best, and potentially obstructing ethical decision making in worse scenarios.

## A revised EMA

Despite its powerful theoretical foundation, McMahan’s EMA may potentially be ethically problematic in the context of dementias, and emerging methods to prevent and treat these conditions. Here, I will show why I think this is the case, and produce a Revised Embodied Mind Account (henceforth ‘REMA’) aiming to better deal with these contexts.

It is worth noting that McMahan explicitly deals with the prospect of dementia—Alzheimer’s disease in particular—to some extent when elaborating on the EMA. This occurs to some degree when discussing the weaknesses of psychological continuity theory. [McMahan, pp. 43–86]^7^ He has also written on the topic in other publications.^37^ However, mainly, his position is declared in the very last section of the very last chapter in his book ‘The Ethics of Killing’, titled ‘The withering away of the self.’^7^

In general, the EMA entails that a person ‘continues to exist throughout the progress of Alzheimer’s disease, until the disease destroys one’s capacity for consciousness.’ [McMahan, p. 65]^7^ To support this, McMahan argues that ‘most people’s intuitive view is that, even in cases of dementia or of radical amnesia and personality change, one continues to exist.’[McMahan, p. 257]^7^ Although it may be true that many people think that we continue to exist throughout even the later stages of dementia, there are at least two problems with McMahan’s assumption. First, data on perception of identity (or ‘self’) persistence in dementia are currently inconclusive.^1^ Second, studies on the topic all use different definitions of selfhood and/or identity, and criteria of measurement.^37 38^ This leaves McMahan’s uncited presumption regarding most people’s intuitions with a fairly weak support. Similarly, Lesser^39^ has argued: ‘people do indeed sometimes talk as if this were the case: ‘He’s not there anymore’ is sometimes said. But it seems fairly clear that, whatever they say, this is not something they literally believe. Indeed, one might suggest that if this were what people really did believe they should be less distressed by the dementia of a relative: it should be easier to accept that a loved person has gone away and been replaced than that they are still here but unable to recognize their family or continue the relationship with them’.

But these (again, unsubstantiated) claims are contradicted by evidence on anticipatory grief in dementia carers, indicating that grief is more prevalent in carers in the months and years prior to the death of the cared-for, than in the months after they have passed.^40–42^ Granted, the inconclusive and disparate results in studies on the subject also go the other way: there is no evidence that most people *don’t* have the intuition that we continue to exist in late-stage dementia, or related neurodegenerative conditions. If anything, there seems to be evidence that intuitions on the matter are quite varied.

In a condensed version, McMahan’s view yields the following result in cases of progressive dementia: (1) Identity is preserved in a person until her brain’s capacity to uphold consciousness ceases—at which point she ceases to exist. (2) The disease gradually diminishes the basis for egoistic concern, consisting in the physical, functional and organisational continuity of the brain. Only physical and functional continuity of the brain is necessary for identity persistence. (3) A person in late-stage dementia (‘Demented Patient’, in McMahan’s terminology) may be regarded as ‘merely a fragment’ of her previous self (‘Patient at Onset’, in McMahan’s terminology). [McMahan, p. 494]^7^ (4) Because of the severed basis for egoistic concern, it would be rational for ‘Patient at Onset’ to care less about herself as ‘Demented Patient’ than she would care about herself was she not to develop dementia.[McMahan, p. 493–496]^7^

In this manner (1–4), the EMA implies that there is a weaker rational basis for a person A to be egoistically concerned about a person B, while there is at the same time a stronger rational basis for A to be egoistically concerned about A, and A=B. This seems odd.^10^ Although this makes sense from an EMA perspective (because the determinator of egoistic concern is not mainly the identity relation), it is less clear that it is a plausible position to argue in applied and clinical ethics. At the very least, it appears puzzling to a point where it is likely to obstruct decision-making procedures involving the ethical value of personal identity. For our purposes, this is quite problematic. I therefore take this objection—call it ‘The Identity-Concern Objection’—as counting against EMA as a theory of personal identity in the context of age-related neural decline and disease.

A second objection worth raising here concerns the status of persons who are suffering from severe neural damage due to dementia and related conditions, and what becomes of them if we manage to save them. To illustrate this point, let us consider a hypothetical case.

### The re-emergent patient

A 65-year-old man, Fenix, is suffering from late stage Alzheimer’s disease, and co-occurring cognitive impairments. Fenix has not been responsive for an extended period of time, and is living a semi-conscious life mostly spent in bed. Gradually, over the years of decline, Fenix’s memories have faded, values changed and attitudes altered to a point where he does not recognise anyone, and his family and loved ones all feel as if he is simply ‘gone’. Nonetheless, after an extensive decision-making procedure including Fenix, his family and a medical team, it has been decided that an experimental treatment should be attempted as a last desperate resort: a new technology for radical neural tissue regeneration has been developed, and approved for human trials. Fenix is going to be one of the first to receive this treatment. After some time, the medical team makes the incredible discovery that the treatment seems to be working! And indeed, bit by bit, the neural cells, connections, and networks of Fenix’s brain regenerate, gradually ‘waking’ Fenix up from his semi-conscious state, and into a conscious and increasingly responsive state. After 3 years of treatment, the medical wonder is completed, and Fenix has similar cognitive abilities to those of other 68 years old. However, as the old neural tissue is forever gone, so are his memories, and previous values, attitudes, interests and his personal relationships are severed.^11^

Note that Fenix’s brain never fully stopped supporting his consciousness throughout this process. Following the line of reasoning of the EMA, we should not only accept that ‘Fenix at onset’ and ‘semi-conscious Fenix’ are the same person, but also that the 68-year-old ‘re-emerged Fenix’ is the same person as the previous two instances of Fenix. In addition, if we go along with McMahan’s account, we should accept that ‘Fenix at onset’ should care very little what happens to him in the two latter stages.^12^ In short: ‘Fenix at onset’ will survive Alzheimer’s disease, but it is irrational of him to care about his surviving self in the way people normally care about their future selves.

Repeating my earlier position regarding this conclusion, it appears counterintuitive at best. If Fenix can survive such a terrible condition and intensive treatment, it seems that he should care about it quite a bit, and that he and his loved ones should celebrate that he is alive and well! However, looking closely at the case, another conclusion appears more likely. Namely, that the identity relation has been broken, and the ‘re-emerged Fenix’ would not be the same person. If this is true, it is certainly true that ‘Fenix at onset’ should not have egoistical concern about ‘re-emerged Fenix’. Not only that, but it is arguably the case that he should dread the existence of a person who—after his own demise—takes its seat in his body, and possibly interacts with his family as if an identity relation was upheld. Certainly, this is the stuff of nightmares!

Now, two things are required: we need to provide a plausible argument for the claim that Fenix does not survive the treatment, and we need to show why that is the case. The two issues are closely intertwined. First, the claim that ‘Fenix at onset’ will not survive to become ‘re-emerged Fenix’ could be supported by the failing of any one, several, or all of the following identity relations: (A) ‘Fenix at onset’ = ‘semi-conscious Fenix’, (B) ‘semi-conscious Fenix’ = ‘re-emerged Fenix’, (C) ’Fenix at onset’ = ‘re-emerged Fenix’. Depending on where the identity relation breaks, our ethical analysis ought to be focused on different issues. For instance, is it the breaking down of the brain to a bare minimum (for consciousness to hold) that causes us to cease to exist, or is it the treatment where we attempt to bring people back? If we assume that ‘semi-conscious Fenix’ truly is stripped of just about everything except the very last functions necessary to uphold some level of consciousness, I am inclined to take the following standpoint:

1. It is more correct to say that ‘Fenix at onset’ is *not* the same person as ‘semiconscious Fenix’, than to say that they are the same person.
2. It is more correct to say that ‘semiconscious Fenix’ is not the same person as ‘re-emerged Fenix’, than to say that they are the same person.
3. ‘Fenix at onset’ is definitely not the same person as ‘re-emerged Fenix’.

Now, this standpoint may look uncomfortably loose for the liking of many personal identity theorists. But it is not too far from previous solutions of other prominent accounts: Parfit’s psychological continuity accepts the existence of prepersons and postpersons, and similarly, in ‘The withering of the self’, McMahan argues that a person in late-stage dementia is to be viewed as ‘a fragment’ of her former self.^7 19^ This standpoint, then, means neither that the person persists, nor that a new person has emerged in her place, but simply that the ‘fragment’ of a person is not sufficient for identity relations to hold. On my view, however, the following holds: when a person has suffered so badly from neurodegeneration that only the fundamental physical capacity to uphold (minimal) consciousness remains—while it is correct that a fragment of the person remains, it is more correct to say ‘that used to be X’ than to say ‘that is X’.

I take the oddness in the McMahanian EMA—the position that we persist throughout the decline, minimally conscious states, and re-emergence processes—comes from the claim that the physical basis for consciousness is the sole determinator of identity. With this, I hope to have shown why I think that it is the case that Fenix does not survive the treatment: something is still missing, for the identity relation to seem plausible. Which leads us immediately to the second question: *why* is it that Fenix does not survive?

The answer to this question is difficult. The core of the crux, I think, is a fundamental mistake in the focus of the enterprise. As argued in the subsection on the narrativist accounts above, there is something to be said about the narrativist critique of traditional philosophical theory on personal identity, in that it asks why we should focus solely on numerical identity.^23–25^ Indeed, although it is clear that we can conceptually differentiate numerical identity (that we are) and qualitative identity (how we are), it is less clear why they should be separated if we want to understand personal identity in dementia. Although I have rejected narrativists’ views as apt accounts for personal identity of over time, I subscribe to this specific point of critique. Unlike narrativist theorists, however, I see no reason why these personality traits, relationships, or values need to fit into a coherent, self-told story, in order for the qualitative identity to be maintained. Furthermore, I do not—at least at this point—take correct, accurate, or accessible memory retention to be a necessary condition for personal identity to hold. In this, I differ from most PC theorists. It seems to me perfectly coherent to truthfully say ‘Fenix doesn’t remember anything’, without implying that the identity relation has been broken somewhere. If anything, I am inclined to think that a Frankfurtian view, appealing to character traits and values which in some sense are to be viewed as being of a ‘higher order’ or ‘truly one’s own’,^43^ would be more apt. Furthermore, it may be controversial to claim that relationships are part of this kind of necessary qualitative identity, and at the same time claim that memories are not. The intuition is that if (many and/or close) relationships are severed, it is a signal that the person has changed into someone unrecognisable and fundamentally different. Similarly, Gillet^44^ and McMillan^43^ have argued for the importance of relationships—the ‘web of interlocutions’—for personal identity. At the same time, it seems that one could connect with loved ones without having access to memories about how the relationship(s) came about. Indeed, in empirical studies, memory loss and distortion has been shown to a have fairly limited impact on perceived identity persistence.^45 46^ Finally, it is not clear to what extent it makes sense to talk about personal identity in dementia without acknowledging the importance of relationships throughout decline and treatment.^28^

In principle, although this position is contradictory to the EMA account as McMahan presents it, it is not incoherent with EM theory as a whole—at least not as far as I can see. The revision is perhaps best understood as an attempt to take the *mind* part of Embodied Mind Theory more seriously. Tentatively, then, on the revised EMA, the position regarding personal identity over time can be defined as follows:

**The Revised Embodied Mind Account (REMA)**

We are essentially embodied minds. The criteria for personal identity persistence over time are (a) physical and minimal functional continuity of the brain, and (b) a retention of personality, values, and relationships of a higher order.

## Towards a unified Embodied Mind Theory

The conundrums of personality, identity and existence that occur in progressive dementia will continue to haunt philosophers until the causes of these conditions are all eradicated. But until then, it is of utmost importance that these concepts can be at least somewhat comprehendible and applicable in dementia care. Many ethical issues in dementia care and treatment, ranging from respecting mundane wishes of persons living with dementia, to advance directives prescribing assisted suicide, rest on these and related notions. In adopting or spreading inaccurate accounts of identity within dementia care and treatment, we risk perpetuating misunderstandings in decision-making processes, and ethically objectionable outcomes in future practices: it is impossible for patients, caregivers and medical professionals to navigate the value trade-offs involved in dementia care and treatment if we do not have an adequate understanding of what those values are—including the value of personal identity over time.

There are many coherent and well-developed accounts of personal identity in the literature, ranging from views stressing the importance of coherence of, and identification with, a personal narrative^20–32^ or the physical body,^47–49^ to accounts emphasising (parts of) the brain,^17 18^ or psychological or mental states and traits.^19 33^ In this paper, I have argued that, while fundamentally one of the most promising accounts of personal identity over time, McMahan’s Embodied Mind Account of personal identity leaves explanatory gaps in how dementia affects our identity. Without taking anything away from the basic principles of Embodied Mind Theory, I have additionally suggested a way forward through which we may unify the quantitative and qualitative aspects of personal identity over time, to generate a more comprehensive account. The aim of this has been to improve our grasp of some of the odd phenomena occurring in dementia, and some of those which are bound to emerge as we develop ways to treat related conditions over the coming decades.

## Data Availability

Thera are no data in this work.

## Footnotes

‘Numerical identity’ is in literature sometimes referred to as ‘quantitative identity’. As the terms are mostly used, I understand them as denoting the same kind of relation.

One reviewer of this paper pointed out that one can conceive of a narrativist theory which does not require that the story is self-told—or told by one spatiotemporally coherent self. This would then raise the question in what way (if at all) the storyteller needs to be related to the person the story is about. Is an identity overlap of sorts required, or can anyone tell the story, as long as they can tell it coherently? This question relates to the claims I make later in this paper about the role of relationships in persistence, although I shall not go so far as to suggest that storytelling could be the sole determinator of personal identity. Nonetheless, it is and interesting angle to a complex problem.

DeGrazia concedes this [DeGrazia, p. 114].^21^

This strongly relates to the Teleportation and Branch-line cases in Parfit.^19^ More about this in the discussion on psychological identity theory below.

Sometimes referred to as ‘Psychological Connectedness’, or simply ‘the Psychological View’.

Or ‘Brain Continuity’, or the somewhat sloppy term ‘Physical Continuity’.

Recalling Parfit’s survival criterion—the holding of facts (1) and (2)—Nagel writes: ‘I actually find Parfit’s picture of *survival* depressing – but that of course is by comparison with what *I* take survival to be. By comparison with Parfitian survival, Parfitian death may not seem so depressing.’[Nagel, p. 224]^31^

That is, a guarantee of a *capacity* for consciousness—not a de facto consciousness.

Two interesting observations can be made here. First, McMahan^7^ himself appears to align with the Brain Identity tradition, in referring to Nagel,^31^ Johnston,^34^ and Unger,^35^ although his account arguably has a heavier focus on the specific physical and functional abilities that *allow* us (the minds) to exist, rather than these abilities being identical to us. Second, it is interesting to note that Parfit, in later years, seems to have been leaning away from his previous position on the matter, and more towards McMahan’s.^36^

Hypothetically, perhaps we could treat this apparent contradiction as an opaque context, much like the relation that Lois Lane is in love with Superman, but not in love with with Clark Kent, even though Superman and Clark Kent is the same person. However, it is unclear what exactly would cause the opaqueness in the case of identity. It is not epistemic, at least not at the level discussed here: we can know quite well what awaits us in progressive dementia, and yet, McMahan would argue, we should hold very little egoistic concern towards ‘Demented Patient’.

As much as the cases of Division, Teletransportation, Deprogramming, and The Cure help uncovering some interesting intuitions, [McMahan, p. 23, 56, 66, 77],^7^ there is little space, and arguably little need, for us to investigate them in greater detail here. Instead, I here provide this little hypothetical case, ‘The Re-Emerging Patient’ to get us to the core issues.

At least as far as egoistic concern goes. McMahan points out that there might be other reasons to value what happens to us [McMahan, p. 496].^7^
